# Evaluation of purine-nucleoside degrading ability and *in vivo* uric acid lowering of *Streptococcus thermophilus* IDCC 2201, a novel antiuricemia strain

**DOI:** 10.1371/journal.pone.0293378

**Published:** 2024-02-22

**Authors:** Dayoung Kim, Jin Seok Moon, Ji Eun Kim, Ye-Ji Jang, Han Sol Choi, Ikhoon Oh

**Affiliations:** Research Laboratories, ILDONG Pharmaceutical Co., Ltd., Hwaseong, Korea; Fonterra Coop / Lebanese University, LEBANON

## Abstract

This study evaluated 15 lactic acid bacteria with a focus on their ability to degrade inosine and hypo-xanthine—which are the intermediates in purine metabolism—for the management of hyperuricemia and gout. After a preliminary screening based on HPLC, *Lactiplantibacillus plantarum* CR1 and *Lactiplantibacillus pentosus* GZ1 were found to have the highest nucleoside degrading rates, and they were therefore selected for further characterization. *S*. *thermophilus* IDCC 2201, which possessed the *hpt* gene encoding hypoxanthine-guanine phosphoribosyltransferase (HGPRT) and exhibited purine degradation, was also selected for further characterization. These three selected strains were examined in terms of their probiotic effect on lowering serum uric acid in a Sprague-Dawley (SD) rat model of potassium oxonate (PO)-induced hyperuricemia. Among these three strains, the level of serum uric acid was most reduced by *S*. *thermophilus* IDCC 2201 (p < 0.05). Further, analysis of the microbiome showed that administration of *S*. *thermophlilus* IDCC 2201 led to a significant difference in gut microbiota composition compared to that in the group administered with PO-induced hyperuricemia. Moreover, intestinal short-chain fatty acids (SCFAs) were found to be significantly increased. Altogether, the results of this work indicate that *S*. *thermophilus* IDCC 2201 lowers uric acid levels by degrading purine-nucleosides and also restores intestinal flora and SCFAs, ultimately suggesting that *S*. *thermophilus* IDCC 2201 is a promising candidate for use as an adjuvant treatment in patients with hyperuricemia.

## Introduction

Uric acid is an end product of the metabolism of purines in the body, and elevated serum uric acid levels have been shown to cause both hyperuricemia and gout [1, 2]. The typical range of uric acid in human plasma is from 4 to 7 mg/dL, with concentrations outside this range considered to be abnormal and referred to as hyperuricemia for elevated levels and hypouricemia for low levels [3]. The presence of long-term excess uric acid at levels above 7 mg/dL tends to lead to the formation of crystalline uric acid deposits on joint and cartilage surfaces. The accumulation of uric acid crystals in joints and soft tissues can cause inflammation and excruciating pain, and it is clinically diagnosed as gout. Although humans do not produce enzymes that degrade uric acid, some bacteria in the intestine can degrade one-third of dietary and endogenous uric acid, so recent hyperuricemia-related studies have focused on analyzing intestinal flora [4–6]. The benefits of probiotics include maintaining gut microbiome composition, regulating immune response, managing metabolic disorders, etc. A number of studies have shown that these beneficial effects are related to the composition of the gut microbiota and the metabolites produced by the microbiota. [7]. Studies have shown that *Lactobacillus gasseri* PA-3 can lower serum uric acid levels by reducing the intestinal absorption of both inosine and adenosine-related compounds [8]. In one clinical trial, 25 patients with hyperuricemia and gout were given yogurt containing *L*. *gasseri* PA-3, which was shown to improve their serum uric acid levels [9]. The strain *Lactiplantibacillus plantarum* DM9218-A isolated from food significantly reduced serum uric acid levels in rats with induced hyperuricemia, and it also showed a preventive effect against hyperuricemia, suggesting its potential utility as an adjuvant treatment for hyperuricemia [10]. Another study demonstrated that the strain *Lactobacillus paracasei* S12 isolated from pickles had the ability to degrade uric acid *in vitro* and *in vivo*, thus revealing its potential to be used in treatments aiming to reduce kidney damage [11]. It has also been reported that the strain *Lactobacillus fermentum* 9–4, which has been confirmed to have the ability to degrade purine-nucleosides *in vitro*, lowers uric acid by degrading inosine and guanosine [12]. Moreover, *Lactiplantibacillus pentosus* P2020 has been reported to reduce renal inflammation by inhibiting inflammation-related signals and to reduce uric acid levels by affecting the expression of proteins involved in uric acid transport [13]. Altogether, the results of these studies demonstrate that probiotics are promising alternative adjuvant treatments for hyperuricemia to improve renal and intestinal function [13, 14].

With this background, in the present work, we screened for and isolated a candidate strain with high purine-degrading activity, and we assessed its anti-hyperuricemia activity in potassium oxonate (PO)-induced hyperuricemia rats. We also used fecal analysis to investigate a possible mechanism for lowering the serum uric acid level.

## Materials and methods

### Isolation, DNA extraction, and identification of strains

In this study, five type strains and 10 strains isolated from traditional Korean fermented foods were used to screen lactic acid bacteria with high purine decomposition activity (Table 1). The specific traditional Korean fermented foods used were kimchi and salted fish, which were homogenized with sterilized phosphate-buffered saline (Gibco, NY, USA) to isolate lactic acid bacteria. Serial dilutions of the homogenate were prepared and cultured on MRS agar (BD, MD, USA) for 24 hours under anaerobic conditions using AnaeroPack™-Anaero (Mitsubishi Gas Chemical Co., Inc., Tokyo, Japan) in an incubator (Jeio Tech, Daejeon, Republic of Korea) at 37°C. The harvested colonies were then subcultured into MRS broth and cultured under the same conditions as described above.

**Table 1:** List of strains used in this study.

Classification	Species	Strain
Type	*Lacticaseibacillus paracasei*	KCTC^1)^ 3510
	*Lactiplantibacillus pentosus*	KCTC 3120
	*Lactiplantibacillus plantarum*	KCTC 3108
	*Lactobacillus gasseri*	KCTC 3163
	*Limosilactobacillus fermentum*	KCTC 3112
Isolates	*Lactiplantibacillus pentosus*	GZ1
	*Lactiplantibacillus pentosus*	GI1
	*Lactiplantibacillus pentosus*	PK-3
	*Lactiplantibacillus pentosus*	YM1
	*Lactiplantibacillus plantarum*	CR1
	*Lactiplantibacillus plantarum*	GDG2
	*Lactiplantibacillus plantarum*	GZM1
	*Lactobacillus gasseri*	PA-3
	*Limosilactobacillus fermentum*	IDCC 3901
	*Streptococcus thermophilus*	IDCC 2201

^1)^ KCTC, the Korean Collection for Type Cultures

For the extraction of genomic DNA, cell cultures were centrifuged in a microcentrifuge CF-10 (DAIHAN Scientific Co., Ltd., Daegu, Republic of Korea) at 13,000 g for 10 minutes, after which cell pellets were extracted using the DNeasy Blood and Tissue kit (Qiagen, Hilden, Germany) according to the manufacturer’s recommendations. The concentration of genomic DNA was then calculated using a DS-11+ spectrophotometer (DeNovix, DE, USA), and the genomic DNA was stored at -20°C until being used as a template.

The extracted genomic DNAs were amplified with a 785F (5′-GGATTAGATACCCTGGTA-3′) and 907R (5′-CCGTCAATTCMTTTRAGTTT-3′) primer pair as well as a 27F (5′-AGAGTTTGATCCTGGCTCAG-3′) and 1492R (5′-TACGGCTACCTTGTTACGACTT-3′) primer pair, and the strains were identified via 16S rRNA gene sequencing, which was performed at Macrogen (Seoul, Republic of Korea) according to internal guidelines.

### Screening of LABs capable of degrading inosine and hypoxanthine

To evaluate the inosine and hypoxanthine assimilating ability, the LAB strain was inoculated in MRS broth and cultured for 24 h at 37°C under anaerobic conditions. To begin, 5 mL of the culture broth was centrifuged in a microcentrifuge CF-10 (DAIHAN Scientific Co., Ltd., Daegu, Republic of Korea) at 4,000 g, 4°C for 10 minutes. Next, the cells were washed twice with 3 mL saline (3M, MN, USA), suspended with 1.25 mM of inosine-hypoxanthine solution in potassium phosphate buffer (0.1 M, pH 7.0), and incubated at 37°C for 60 minutes with shaking at 120 rpm. The supernatant sample was then harvested using centrifugation at 4,000 g, 4°C for 10 minutes. After filtering using a 0.45-μm Millipore filter (Whatman, Maidstone, England), the filtrate was measured using an HPLC-UV system according to the protocol described by Law et al. [15]. The 10 μL filtrate was injected into the HPLC system equipped with a Waters T3 column (5 um, 250 × 4.6 mm) connected to an HPLC pump (Alliance e2695; Waters Corp., Massachusetts, USA) and a Waters 2998 UV detector. The column temperature was maintained at 25°C; mobile phase A was 0.1% phosphoric acid in water whereas mobile phase B was acetonitrile. The gradient program was as follows: 0–10 min, 98% A isocratic; 10–15 min, 98% A to 85% A; 15–25 min, 85% A to 20% A; and 25–30 min, 98% A isocratic at a flow rate of 0.8 mL/min. Inosine and hypoxanthine were detected at 254 nm with the respective retention times of 21.8 and 11.8 minutes, and they were quantified through comparison with the standard.

Each compound was quantified based on the peak area value of the HPLC chart. The resolution of inosine-hypoxanthine was measured and calculated according to a previously described research method [16]. The following formula was used to calculate the degradation rate: degradation rate (%) = 100 - ((peak area at 1 hour / peak area at 0 hour) × 100).

### Purine metabolism related gene

**Fig 1:**
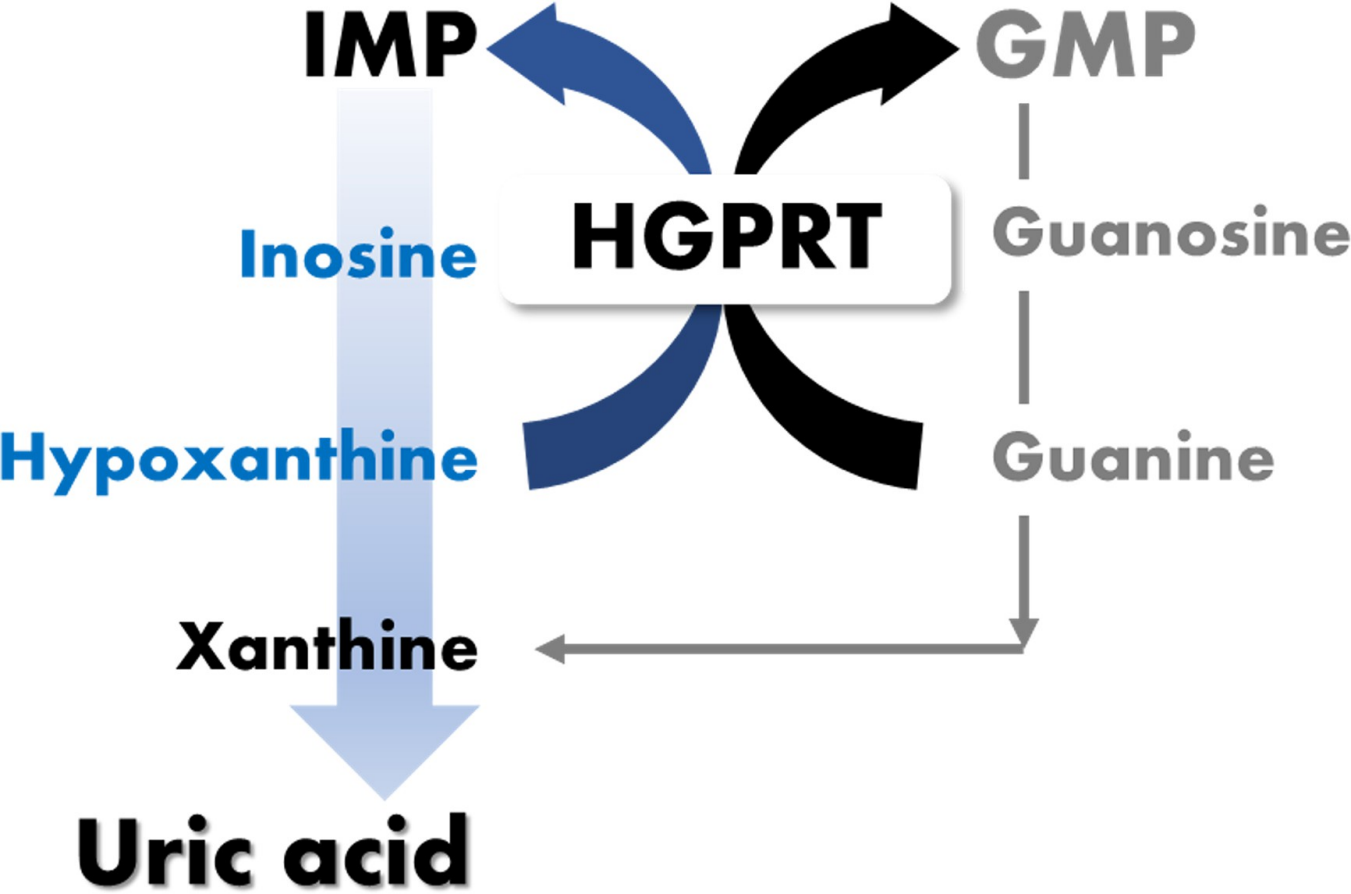
Overview of purine metabolism by HGPRT. The purine metabolic system represents a pathway by which hypoxanthine and guanine can be salvaged by HGPRT. HGPRT serves to catalyze the salvage synthesis of IMP and GMP from the purine bases hypoxanthine and guanine. HGPRT also prevents the accumulation of substrates that are converted to uric acid.

The enzyme hypoxanthine-guanine phosphoribosyltransferase (HGPRT), which lowers uric acid levels by converting hypoxanthine and guanine to guanosine monophosphate (GMP) and inosine monophosphate (IMP), was selected as an important factor [17] (Fig 1). For *S*. *thermophilus* LMG 18311 and *S*. *thermophilus* CNRZ 1066, the *hpt* gene that encoded this enzyme was searched for in the United Protein Database (UniProt: https://www.uniprot.org) database.

### Experimental design of candidate strains of probiotics

The animal experiment in this study was performed at the Dt&CRO Efficacy Evaluation Center (Yongin, Republic of Korea) with approval from the Institutional Animal Care and Use Committee (IACUC approval number: 210317). To begin, 54 male Sprague-Dawley (SD) rats (8-week-old) were obtained from Orientbio Inc. (Seongnam, Republic of Korea) and adapted to the environment at 22 ± 3°C and 50 ± 20% humidity for 7 days with a 12-hour light/dark cycle. Animals were fed solid food formulated for laboratory animals (Teklad Certified Irradiated Global 18% Protein Rodent Diet 2918C, Envigo RMS, Inc., Indianapolis, IN, USA) and tap water that was filtered using a water filter sterilizer and then irradiated with ultraviolet rays; both were freely available for consumption. After 7 days of acclimatization, rats were randomly assigned to nine groups with six rats in each group: (a) normal group (G1); (b) hyperuricemia group (G2); (c) *L*. *plantarum* CR1 (1×10^8^ CFU/ day, G3); (d) *L*. *pentosus* GZ1 (1×10^8^ CFU/ day, G4); (e) *S*. *thermophilus* IDCC 2201 (1×10^8^ CFU/ day, G5); (f) *L*. *plantarum* CR1+ *S*. *thermophilus* IDCC 2201 (1×10^8^ CFU/ day, G6); (g) *L*. *pentosus* GZ1+ *S*. *thermophilus* IDCC 2201 (1×10^8^ CFU/ day, G7); (h) *L*. *gasseri* PA-3 (1×10^8^ CFU/ day, G8); and (i) Allopurinol (50 mg/kg rat/day, G9). Test substances and allopurinol were administered orally. The normal group was intraperitoneally administered a dose of 8 mL/kg of 0.5% carboxy methyl cellulose (Sigma-Aldrich, MO, USA) that served as an excipient. The hyperuricemia induction and test substance administration groups were intraperitoneally administered a dose of 8 mL/kg (250 mg/kg) PO (TCI, Tokyo, Japan) for 7 days. Test materials were administered 1 hour after PO administration. On day 7, prior to animal sacrifice, blood was collected from the tail vein, and biochemical indicators such as blood uric acid were measured using a blood biochemical analyzer (Hitachi Ltd., Tokyo, Japan). To analyze the intestinal microflora, fecal samples were collected from the animals on the day of test termination and stored at -80°C.

### Experimental design of *S*. *thermophilus* IDCC 2201 by concentration

The *S*. *thermophilus* IDCC 2201 concentration-dependent animal experiment was performed in the same manner as described above. The animal experiment was approved by IACUC (approval number: 220203) and conducted at the Dt&CRO Efficacy Evaluation Center. For this experiment, 40 male SD rats (8-week-old) were obtained and randomly assigned to five groups with eight rats each. The test material was prepared as follows: To begin, *S*. *thermophilus* IDCC 2201 was inoculated at a concentration of 1% (v/v) into Lactobacilli MRS broth (BD, MD, USA) and then cultured overnight under anaerobic conditions using AnaeroPack™-Anaero (Mitsubishi Gas Chemical Co., Inc., Tokyo, Japan) in an incubator (Jeio Tech, Daejeon, Republic of Korea) at 37°C. Next, 1 mL of overnighted cultivation liquid was inoculated into MRS broth supplemented with 1.3 mM hypoxanthine, and this was allowed to grow at 37°C for 16 hours. The *S*. *thermophilus* IDCC 2201 bacterial cells were obtained by centrifugation (Hitachi, Tokyo, Japan) at 7,000 rpm for 5 min at 4°C, after which they were washed with saline (3M, MN, USA). Test substances for oral administration were prepared by suspending the cells in PBS pH 7.4 (Gibco, NY, USA) supplemented with 20% (w/w) glycerol (Duksan, Ansan, Korea). The substances were prepared at concentrations of 10^7^, 10^8^, and 10^9^ CFU/mL, respectively. The groups were as follows: (a) normal group (G1); (b) hyperuricemia group (G2); (c) *S*. *thermophilus* IDCC 2201-SH (1×10^9^ CFU/day, G3); (d) *S*. *thermophilus* IDCC 2201-SM (1×10^8^ CFU/day, G4); (e) *S*. *thermophilus* IDCC 2201-SL (1×10^7^ CFU/day, G5).

### Fecal samples analysis

#### Fecal DNA purification and metagenome sequencing for gut microbiome analysis

For metagenome analysis, fecal DNA was extracted using the ExgeneTM Stool DNA Mini Kit (GeneAll Biotechnology, Seoul, Republic of Korea) while following the manufacturer’s protocol. The V3-V4 region of the bacterial 16S rRNA gene was amplified using barcoded universal primers, 341F (5’-CCTACGGGNGGCWGCAG-3’) and 805R (5’-GACTACCAGGGTATCTAATC-3’). Microbiome profiling was conducted on the 16S-based Microbial Taxonomic Profiling platform offered by EzBioCloud Apps (ChunLab Inc., Seoul, Republic of Korea).

#### HPLC analysis of short-chain fatty acids (SCFAs) in fecal samples

SCFAs in fecal samples were quantified using HPLC analysis [18]. To extract SCFAs from the fecal samples, 1.0 g of each fecal sample was thawed, suspended in 8 mL of sterile deionized water (Bioneer Corp., Daejeon, Republic of Korea), and homogenized using a vortex mixer for 5 minutes. Next, the solution was centrifuged at 9500 rpm for 10 minutes at 4°C. The resulting supernatant was then filtered through a 0.2-μm cellulose acetate/surfactant-free membrane filter, after which 10 μL was injected into a Waters e2695 HPLC system equipped with a Waters 2998 UV detector. Lastly, chromatographic separation was conducted under isocratic elution conditions using a Concise coregel 87H3 column (7.8 × 300 mm, 5 μm) (Concise Separations, CA, USA) and a mobile phase of 5 mM sulfuric acid, with the detection wavelength set at 210 nm. The other chromatographic conditions included a column oven temperature of 35°C, a flow rate of 0.6 mL/min, and a run time of 60 minutes. Following chromatographic separation, individual SCFAs (acetic acid, propionic acid, and butyric acid) were identified and quantified based on the standard retention times and peak areas (Sigma-Aldrich, MO, USA).

### Pangenome analysis and strain specific gene extraction

After the *in vivo* experiment, the BPGA (Bacterial Pan Genome Analysis tool) v.1.3 computational pipeline was used to extract strain-specific genes for the selected *S*. *thermophilus* IDCC 2201 [19]. To this end, seven annotated *S*. *thermophilus* genome sequences were used as input files (Table 2). The unique genome database was constructed with a 50% identity cutoff using the USEARCH algorithm [20]. The strain-specific candidate gene for *S*. *thermophilus* IDCC 2201 was confirmed to be specific for the strain analyzed through the basic local alignment search tool (BLAST). Genes with high target specificity were selected, and primers were designed using the PCR Primer Design Tool (Eurofins Genomics, Ebersberg, Germany) while considering amplicon size and GC content.

**Table 2:** List of seven genome sequences analyzed in this study.

Species	Strain	Size (Mb)	GC%	Proteins	Assembly level	Accession no.
*Streptococcus thermophilus*	IDCC2201	1.79484	39.2	1588	GCA_004114735.1	CP035306.1
*Streptococcus thermophilus*	ND03	1.83195	39	1629	GCA_000182875.1	CP002340.1
*Streptococcus thermophilus*	MN-BM-A02	1.85043	39	1630	GCA_001008015.1	CP010999.1
*Streptococcus thermophilus*	KLDS SM	1.85679	39.1	1614	GCA_001663795.1	CP016026.1
*Streptococcus thermophilus*	KLDS 3.1003	1.89996	38.9	1674	GCA_001705585.1	CP016877.1
*Streptococcus thermophilus*	DGCC 7710	1.85121	39	1624	GCA_002843115.1	CP025216.1
*Streptococcus thermophilus*	NCTC12958	2.10227	39	1888	GCA_900474985.1	LS483339.1

### Strain-specific primer specificity and quantitative evaluation

Real-time PCR was performed using the CFX96 Deep Well Real-time System (Bio-Rad, CA, USA). The PCR mixture consisted of 20 ng of template DNA, 10 pmol/μL of primer pairs, 10 μL of 2×SYBR qPCR Mix (Toyobo, Osaka, Japan), and deionized distilled water to a total volume of 20 μL. Real-time PCR proceeded as follows: Initial denaturation was initiated at 95°C for 2 min, followed by 40 cycles of 95°C for 5 sec and 60°C for 30 sec. A melting curve was obtained from 65°C to 95°C with holding and heating for 15 seconds at each step [21]. For the quantification of *S*. *thermophilus* IDCC 2201 in fecal samples, a standard curve was constructed with the extracted *S*. *thermophilus* IDCC 2201 genomic DNA. After being incubated at 37°C for 48 hours, *S*. *thermophilus* IDCC 2201 was cultured on MRS agar plates and counted to confirm that the number of viable cells was 10^9^ CFU/mL. Then, genomic DNA was extracted and diluted to 10^2^ CFU/mL in deionized water using the serial decimal dilution method. Fecal samples were quantified by Log (CFU/g) based on the established standard curve.

### Statistical analyses

All experimental data are presented in the form of mean ± standard deviation. Differences between the control and experimental groups were analyzed by two-sample t-test or Kruskal–Wallis test to determine statistical significance. All statistical analyses were performed using GraphPad Prism software (version 8.0). A between group p-value of 0.05 or less was considered to indicate a statistically significant difference.

## Results & discussion

### *In vitro* for screening probiotic candidate strains

#### Purine metabolism-related gene and analysis of hypoxanthine degrading

Among the different types of strains listed by the Ministry of Food and Drug Safety (MFDS, Cheongju, Republic of Korea), the *hpt* gene was identified in the *S*. *thermophilus* strain. In particular, the *hpt* gene of *S*. *thermophilus* LMG 18311 was identified as UniProt ID: Q5M6K8, while the *hpt* gene of *S*. *thermophilus* CNRZ 1066 was identified as UniProt ID: Q5M216. The *S*. *thermophilus* IDCC 2201 genome possessed the gene locus_tag = ESP48_05170 en-coding HGPRT. This protein is one of the essential enzymes in the purine salvage pathway that catalyzes the magnesium-dependent formation of IMP and GMP [22]. It is also involved in the reduction of uric acid levels by recovering purines that have been formed from the conversion of hypoxanthine and guanine to IMP and GMP, respectively [23, 24]. This result suggests that *S*. *thermophilus* IDCC 2201 can reduce uric acid levels through purine metabolism.

Based on the properties described above, the ability of *S*. *thermophilus* IDCC 2201 to degrade hypoxanthine was evaluated through HPLC. As a result, *S*. *thermophilus* IDCC 2201 was shown to degrade 48.48 ± 0.04% of 1.25 mM hypoxanthine for 1 hour. The development of strains that degrade purine compounds, such as hypoxanthine, has emerged as a potential treatment pathway for hyperuricemia [25]. Uric acid reduction through the inhibition of hypoxanthine has been confirmed to be a potential route for improving hyperuricemia.

#### Screening of LABs capable of degrading inosine

**Fig 2:**
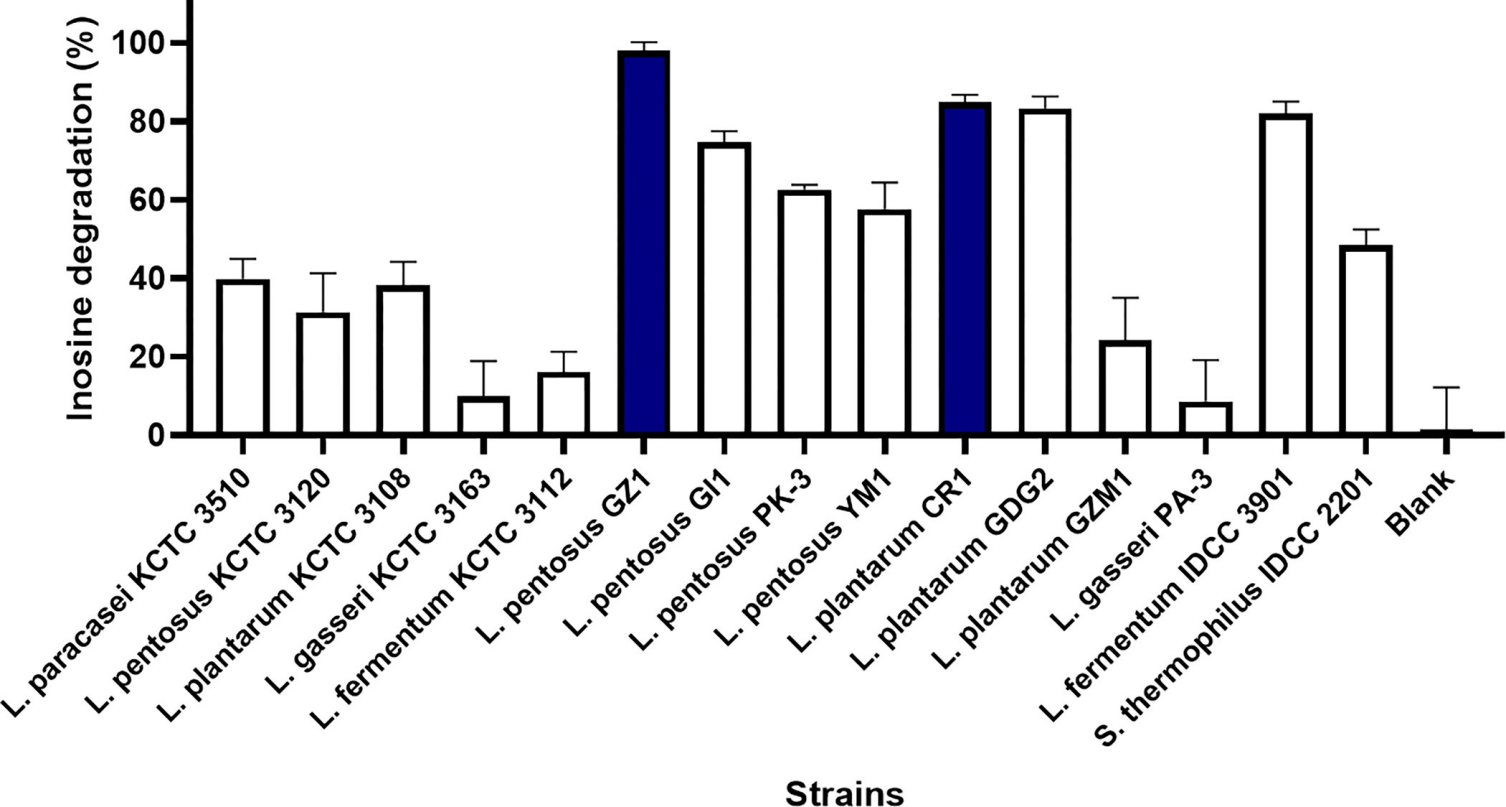
Inosine degradation analysis by HPLC.

For the selection of probiotics candidate strains, 15 candidate strains were evaluated for inosine degradation based on HPLC analysis. The degradation rate was calculated using the degradation rate formula, and the results are shown in Fig 2. Among the 15 strains tested, the two strains with the highest inosine degradation rates were selected: *L*. *plantarum* CR1, in which 1.25 mM inosine was degraded to 84.65% inosine, and *L*. *pentosus* GZ1, in which 1.25 mM inosine was degraded to 99.98% inosine. Inosine is an intermediate metabolite of purine metabolism that is related to the production of uric acid, which causes hyperuricemia [26]. The inosine degradation rates of the strains that have been selected in previous studies have been confirmed to range from 67.59% to 100% [16]. The inosine degrading abilities of the two strains selected in this study were 84.65% and 99.98%, which was confirmed to be similar to the values presented in a previous study. *S*. *thermophilus* IDCC 2201, which has a gene related to purine metabolism and has been confirmed to exhibit hypoxanthine degrading ability, showed a degradation of 50.12% for inosine. *L*. *plantarum* CR1 and *L*. *pentosus* GZ1, which both have excellent inosine degradation, underwent additional testing to further evaluate their hypoxanthine degrading abilities, but they showed degradation rates of 1.42 ± 2.05% and 0.69 ± 1.13%, respectively (S1 Fig).

After analyzing the gene related to purine metabolism and confirming purine degradation, three strains were ultimately selected: *S*. *thermophilus* IDCC 2201, *L*. *plantarum* CR1, and *L*. *pentosus* GZ1. Subsequent *in vivo* assays were performed using these three strains screened *in vitro*.

### Efficacy evaluation of probiotics in rats with hyperuricemia

**Fig 3:**
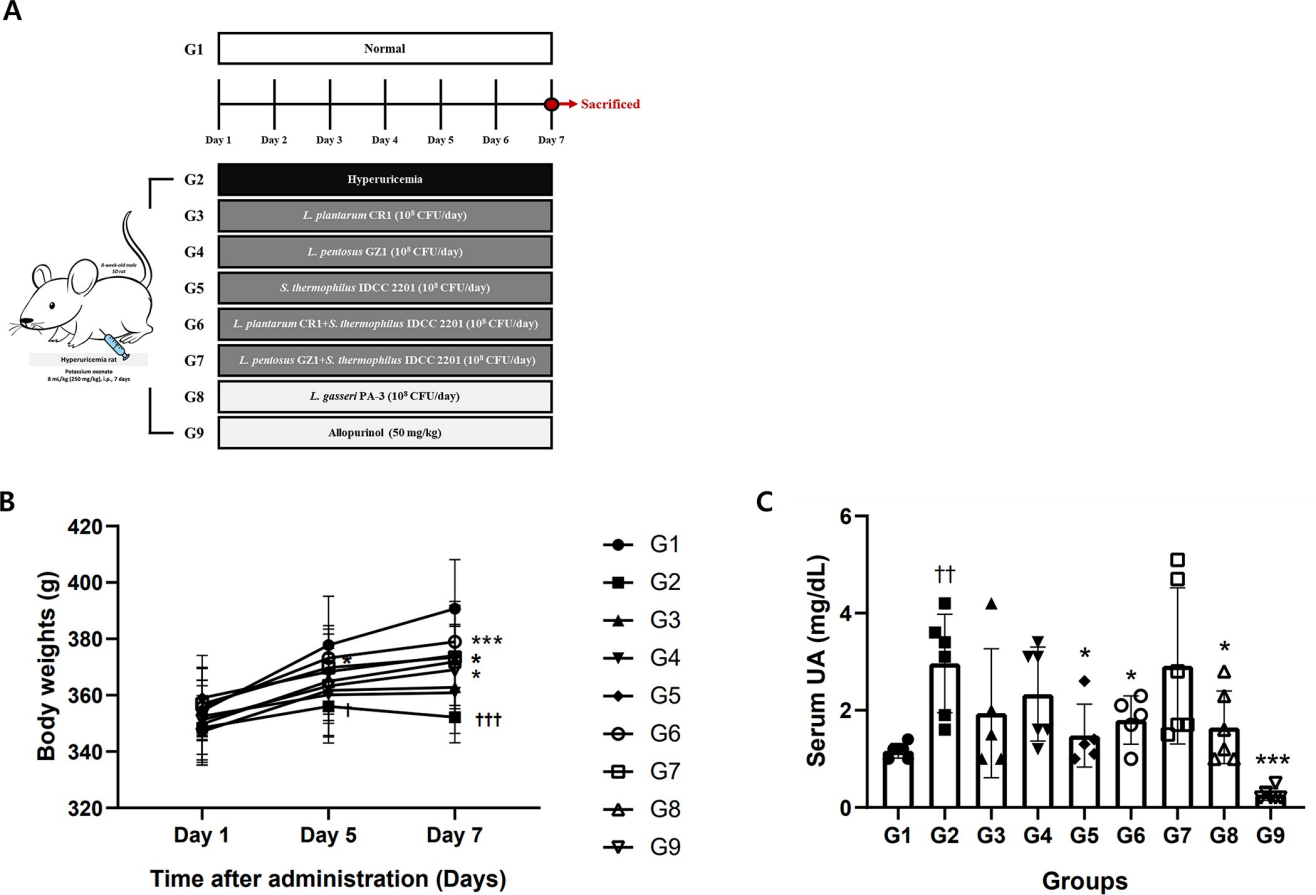
The effect of probiotics on the reduction of blood uric acid level in hyperuricemia rats. (A) Schematic depiction of the po-induced hyperuricemia model. (B) Body weights (g). (C) Serum UA (mg/dL). On the last day of administration of the test substance, 3 hours after administration of the test substance or the comparative substance, blood was collected from the jugular vein of the rat, while the uric acid level was measured using a blood biochemical analyzer. G1: normal group; G2: hyperuricemia group; G3: *L*. *plantarum* CR1 (1×10^8^ CFU/ day); G4: *L*. *pentosus* GZ1 (1×10^8^ CFU/ day); G5: *S*. *thermophilus* IDCC 2201 (1×10^8^ CFU/ day); G6: *L*. *plantarum* CR1+*S*. *thermophilus* IDCC 2201 (1×10^8^ CFU/ day); G7: *L*. *pentosus* GZ1+ *S*. *thermophilus* IDCC 2201 (1×10^8^ CFU/ day); G8: *L*. *gasseri* PA-3 (1×10^8^ CFU/ day); and G9: Allopurinol (50 mg/kg rat/day). Data are presented as mean ± SD values (N = 6). Different symbols indicate significant differences according to the results of statistical analysis using a t-test. ^†^ p < 0.05, ^††^ p < 0.01, ^†††^ p < 0.001 vs G1, * p < 0.05, *** p < 0.001 vs G2.

In the *in vitro* experiments, *S*. *thermophilus* IDCC 2201, which was tested for hypoxanthine degradation, and *L*. *plantarum* CR1 and *L*. *pentosus* GZ1, which were tested for inosine degradation, all showed high degradation rates. These three selected species were used in animal experiments in which hyperuricemia was induced. SD rats with hyperuricemia induced by PO were used to study the effect of improving hyperuricemia [16]. PO, which increases blood uric acid level by inhibiting uricase, was used to examine the effect of candidate strains *L*. *plantarum* CR1, *L*. *pentosus* GZ1, and *S*. *thermophilus* IDCC 2201 on blood uric acid level in hyperuricemia rats, and it was intraperitoneally administered for 7 days to induce hyperuricemia. Moreover, LAB and allopurinol were orally administered 1 hour after PO administration for 7 days (Fig 3A). During the experimental period, body weight increased in a statistically significant manner compared to G2 in all groups except for the G3 and G4 administration groups (Fig 3B). The most important indicator of hyperuricemia is the uric acid content in the blood. As shown in Fig 3C, the average level of uric acid in G2, which caused hyperuricemia, was 2.97 ± 1.01 mg/dL, which was more than double the average level of 1.17 ± 0.15 mg/dL in G1 in a statistically significant increase. Meanwhile, the uric acid levels were significantly decreased in the positive control groups, G8 and G9 (p < 0.05, p < 0.001). Altogether, these markers indicate that the hyperuricemia model was successfully established.

G5 and G6 significantly decreased uric acid levels by 1.48 ± 0.65 mg/dL and 1.8 ± 0.50 mg/dL, respectively (p < 0.05). Compared to the G2 group, the blood uric acid levels in the G5 and G6 administration groups were significantly reduced by 50.11% and 39.33%, respectively. These are effective values compared to those that were previously reported for *L*. *fermentum* NCUH003018 (30.77%) [16].

### Effects of *S*. *thermophilus* IDCC 2201 on the composition of gut microbiota in rats with hyperuricemia

**Fig 4:**
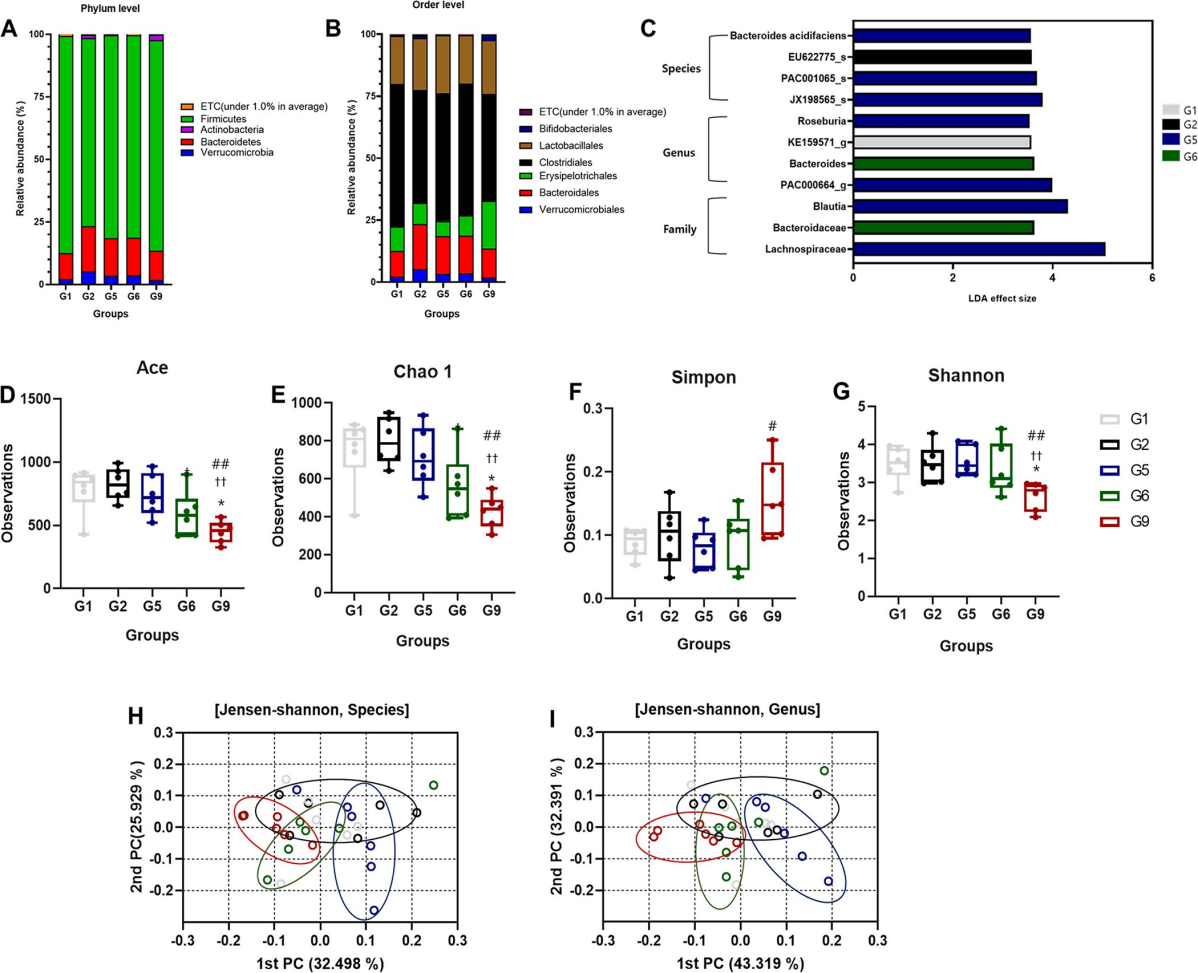
Effects of *S*. *thermophilus* IDCC 2201 on gut microbiota in SD rats with PO-induced hyperuricemia. Distribution of gut microbiota at the (A) phylum and (B) order levels. (C) Taxonomic levels from the phylum to the genus (LDA score > 3.5, p < 0.05). Horizontal bars represent the effect size for each taxon. LDA, linear discriminant analysis. Boxplot of (D) Ace, (E) Chao1 index, (F) Simpson index, and (G) Shannon index. Beta diversity plot of principal coordinate analysis (PCoA). PCoA was performed at a (H) species and (I) genus level with Jensen-Shannon distances, including unclassified operational taxonomic units. G1: normal group; G2: hyperuricemia group; G5: *S*. *thermophilus* IDCC 2201 (1×10^8^ CFU/ day); G6: *L*. *plantarum* CR1+*S*. *thermophilus* IDCC 2201 (1×10^8^ CFU/ day); and G9: Allopurinol (50 mg/kg rat/day). A significant difference from G1 is denoted as * p < 0.05, a significant difference from G2 is denoted as ^†^ p < 0.05 and ^††^ p < 0.01, and a significant difference from G5 is denoted as ^#^ p < 0.05 and ^##^ p < 0.01.

Fig 4 depicts the effect of *S*. *thermophilus* IDCC 2201 on the gut microbiota of SD rats with PO-induced hyperuricemia. Among the experimental categories of interest, the investigation discovered conspicuous fluctuations in microbial composition at the phylum level. However, these fluctuations were not statistically significant (Fig 4A). By contrast, the allopurinol-treated group (G9) showed a statistically significant increase in the relative abundance of *Erysipelotrichaceae* at the order level compared to both G5 (p < 0.05) and G6 (p < 0.05) (Fig 4B). *Erysipelotrichaceae*, which is a family of bacteria found in the gut microbiota of humans and animals, has been linked with several health problems including cancer and gastrointestinal disorders [27].

The changes in the G5 group’s gut microbiota can be linked to the therapy for hyperuricemia and gout by specific mechanisms. The treatment with *S*. *thermophilus* IDCC 2201 significantly boosted the *Lachnospiraceae* family (LDA effect size = 5.059, p = 0.002). Significant improvements were also observed in the *Blautia* genus (LDA effect size = 3.798, p = 0.036), *Roseburia* genus (LDA effect size = 3.537, p = 0.010), and *Bacteroides acidifaciens* species (LDA effect size = 3.564, p = 0.034) within Group G5. The increase in the population of the *Lachnospiraceae* family indicates that *S*. *thermophilus* IDCC 2201 strain therapy could have uniquely affected this family, which is known for its health benefits, such as the production of SCFAs and anti-inflammatory effects [28]. The rise in *Blautia* and *Roseburia* species could support the anti-hyperuricemic effects of *S*. *thermophilus* IDCC 2201. These species are recognized for their function of manufacturing butyrate, which assists in conserving intestinal barrier roles and reducing inflammation [29]. The analysis of the diversity of gut microbiota, as measured via the Ace (Fig 4D) and Chao1 (Fig 4E) indices, revealed a significant reduction within Group G9 in comparison to Groups G1 (p < 0.05), G2 (p < 0.01), and G5 (p < 0.01). Noticeable differentiation could also be observed between Groups G2 and G6 (p < 0.05), while the Simpson index (Fig 4F) displayed a sharp contrast between Groups G5 and G9 (p < 0.05). Remarkably, the Shannon index (Fig 4G) indicated a significant difference between Groups G9 and G1 (p < 0.05), G2 (p < 0.01), and G5 (p < 0.01). The diversity of gut microbiota underwent significant changes in response to various treatment groups, implying the potential utility of these treatment groups in treating hyperuricemia.

PCA was utilized to evaluate the diversity of gut microbiota at the species (Fig 4H) and genus (Fig 4I) levels while employing Jensen-Shannon distances. There was a notable difference found in the composition of gut microbiota between Groups G9 and Groups G2 (p = 0.012, 0.025), G5 (p = 0.018, 0.016), and G6 (p = 0.042, 0.011). These results suggest that there was a significant difference in gut microbiota composition between the treatment groups that received allopurinol.

It can therefore be concluded from these findings that the administration of *S*. *thermophilus* IDCC 2201 (G5) had a substantial impact on the diversity and composition of gut microbiota in SD rats with PO-induced hyperuricemia. Noticeable variations were observed among the experimental groups, indicating the need for further investigation. Interestingly, allopurinol therapy also led to noticeable alterations in the structure or diversity of the gut microbiota compared to the gut microbiota seen in hyperuricemia induced by PO and the cohorts receiving probiotics.

### Uric acid reduction effect of *S*. *thermophilus* IDCC 2201 by concentration in hyperuricemia rats

**Fig 5:**
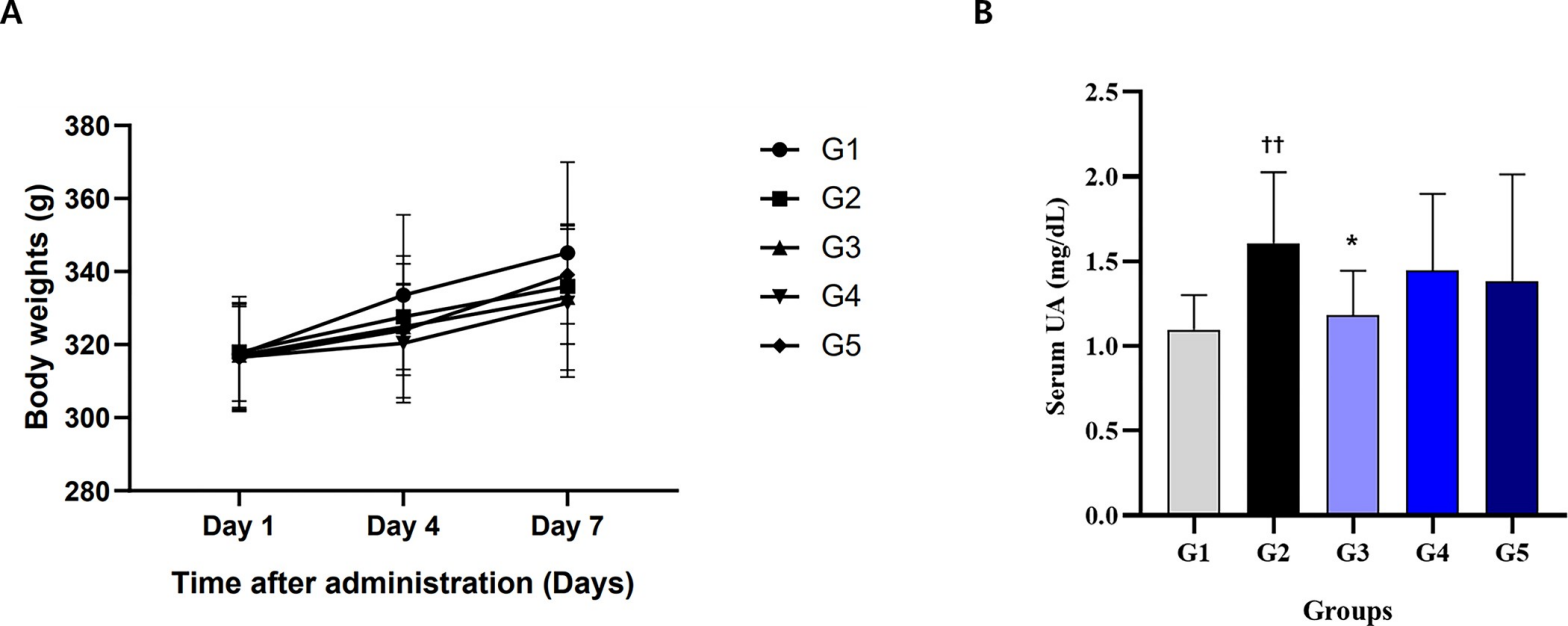
Effects of *S*. *thermophilus* IDCC 2201 concentrations on the reduction of blood uric acid levels in hyperuricemia rats. (A) Body weights (g). (B) Serum UA (mg/dL). On the 7th day of the test, 3 hours after administration of the test substance or the comparative substance, blood was collected from the animal’s jugular vein for measurement of the uric acid level. G1: normal group; G2: hyperuricemia group; G3: *S*. *thermophilus* IDCC 2201-SH (1×10^9^ CFU/day); G4: *S*. *thermophilus* IDCC 2201-SM (1×10^8^ CFU/day); G5: *S*. *thermophilus* IDCC 2201-SL (1×10^7^ CFU/day). Data are presented in the form of mean ± standard deviation values (N = 8). Different symbols indicate statistically significant differences according to the results of statistical analysis using t-testing. ^††^ p < 0.01 vs G1, * p < 0.05 vs G2.

Based on the results derived from ‘Efficacy evaluation of probiotics in rats with hyperuricemia’, the *S*. *thermophilus* IDCC 2201 strain was evaluated while focusing on the reproducibility of uric acid reduction in the same animal model as well as dose dependency. There were no statistically significant differences between the weights of any test groups, while the average of G2 was 1.61 ± 0.42 mg/dL, which was a statistically significant increase compared to the average of 1.10 ± 0.20 mg/dL of G1 (p < 0.01) (Fig 5A). This indicates that the animal model was successfully established as intended. To confirm concentration dependence, *S*. *thermophilus* IDCC 2201 was administered at concentrations of 1×10^9^ CFU/day (*S*. *thermophilus* IDCC 2201-SH, G3), 1×10^8^ CFU/day (*S*. *thermophilus* IDCC 2201-SM, G4), and 1×10^7^ CFU/day (*S*. *thermophilus* IDCC 2201-SL, G5), respectively. Group G3 was administered 1.18 ± 0.26 mg/dL, which was statistically significantly lower than that administered to G2 (p < 0.05) (Fig 5B). The G4 and G5 administration groups exhibited respective values of 1.45 ± 0.45 mg/dL and 1.38 ± 0.63 mg/dL, thus showing no significant difference from G2. This indicates that the administration of a high concentration of *S*. *thermophilus* IDCC 2201 has the effect of reducing uric acid in hyperuricemia.

### Analysis of the effect of *S*. *thermophilus* IDCC 2201 on SCFAs in fecal samples

**Fig 6:**
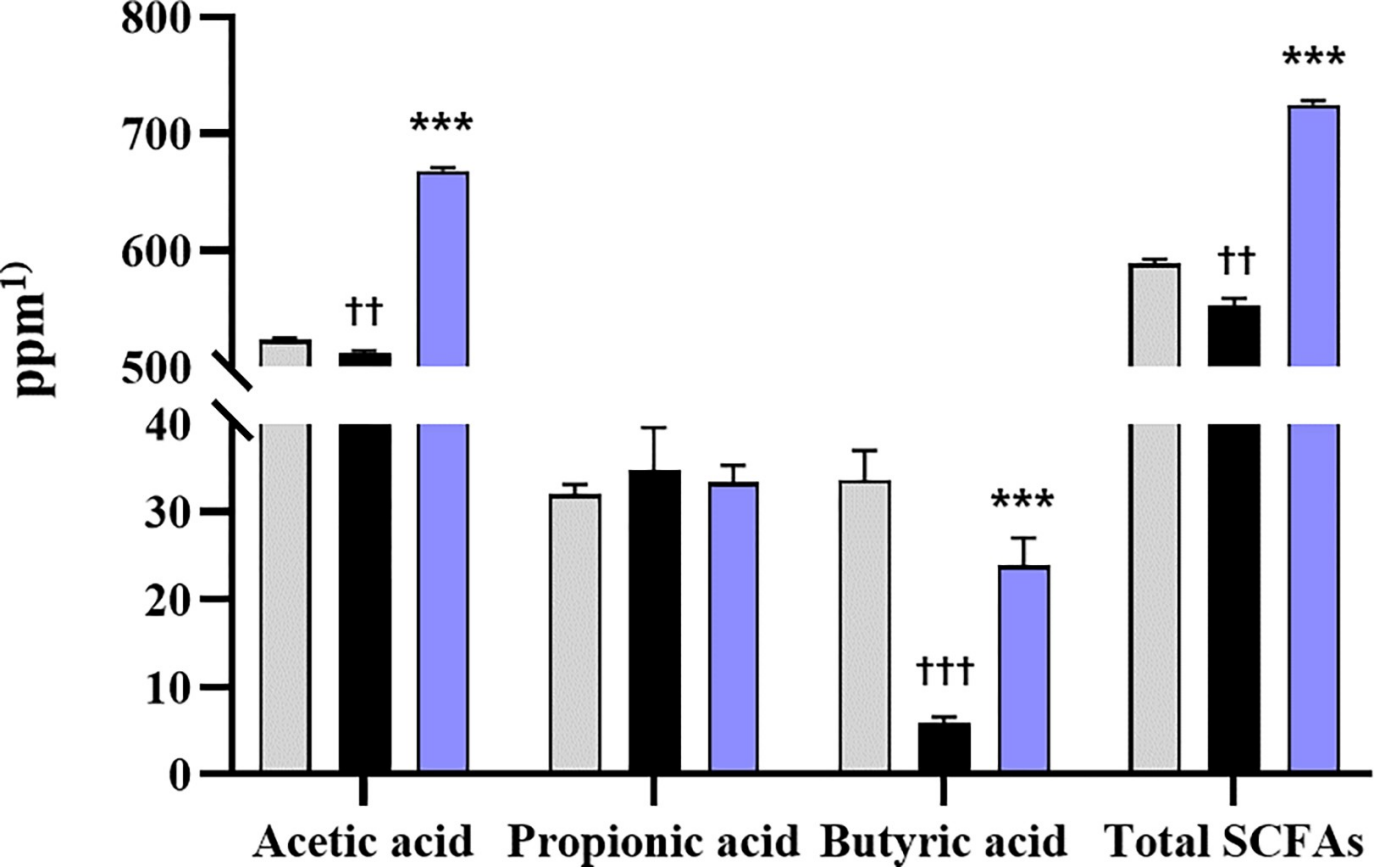
SCFAs content in rat feces. G1: normal group; G2: hyperuricemia group; G3: *S*. *thermophilus* IDCC 2201-SH (1×10^9^ CFU/day). The mean ± standard deviation values (N = 8) are depicted by bars. Different symbols indicate statistically significant differences according to the results of statistical analysis using t-testing. ^††^ p < 0.01, ^†††^ p < 0.001 vs G1, *** p < 0.001 vs G2. ^1)^ ppm = μg/mL.

SCFAs were determined by HPLC in the fecal samples of the rats described in ‘Uric acid reduction effect of *S*. *thermophilus* IDCC 2201 by concentration in hyperuricemia rats’. As shown in Fig 6, G2 showed significantly lower levels of both acetic acid (p < 0.01) and butyric acid (p < 0.001) compared to G1. By contrast, G3 showed significantly higher levels of both acetic acid and butyric acid compared to G2 (p < 0.001, p < 0.001, respectively). Regarding the total content of SCFAs, there was a significant difference between G2 and G3 with p < 0.001.

A previous study reported that hyperuricemia-induced rats have low intestinal bacterial abundance, which results in low concentrations of SCFAs and eventually leads to intestinal barrier dysfunction and increased intestinal permeability [30, 31]. Moreover, in a mouse model of hyperuricemia, SCFAs have been shown to be involved in uric acid metabolism as well as the intestinal barrier [32]. Butyrate has been reported as having a particularly beneficial effect on uric acid excretion by supplying ATP to the cells of the intestinal wall, thus improving uric acid metabolism and helping alleviate hyperuricemia [33, 34]. Therefore, *S*. *thermophilus* IDCC 2201 at a concentration of 1 × 10^9^ CFU/day might contribute to uric acid reduction by supporting SCFAs that have been decreased due to hyperuricemia.

### Analysis of *S*. *thermophilus* IDCC 2201 using strain specific primer

**Fig 7:**
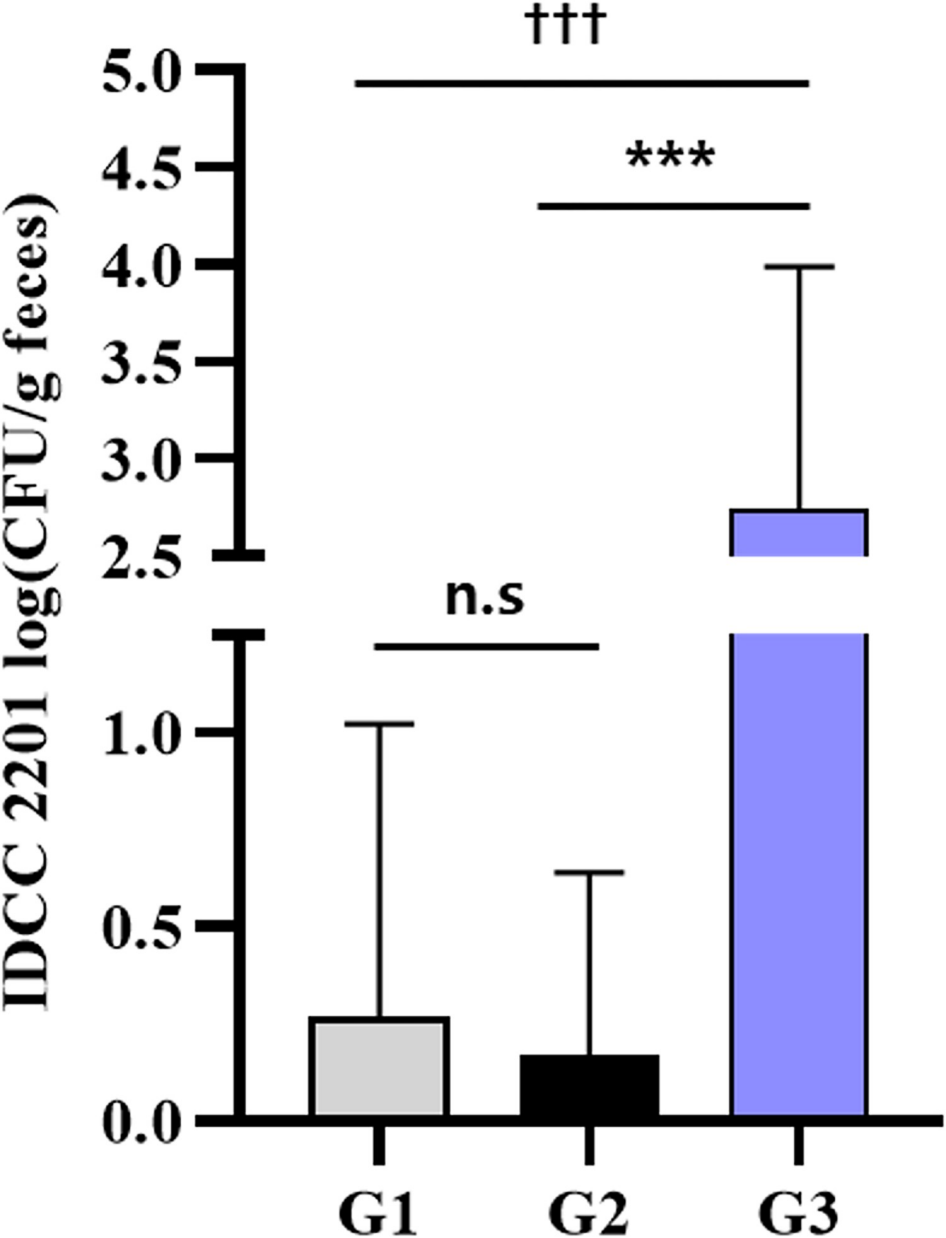
Quantification of *S*. *thermophlius* IDCC 2201 in fecal samples of rats with hyperuricemia administered with *S*. *thermophilus* IDCC 2201, as analyzed by real-time PCR. G1: normal group; G2: hyperuricemia group; G3: *S*. *thermophilus* IDCC 2201-SH (1×10^9^ CFU/day). Data are presented in the form of mean ± standard deviation values (N = 8). Different symbols indicate statistically significant differences according to the results of statistical analysis using t-testing. n.s.: no significant difference between groups. ^†††^ p < 0.001 vs G1, *** p < 0.001 vs G2.

When using the 16S sequencing method, it is difficult to conduct quantitative evaluations at the bacterial species level [35]. To specifically detect the *S*. *thermophilus* IDCC 2201 strain, real-time PCR analysis was performed using the flippase (Accession no. QAU28922.1) gene as a specific marker for *S*. *thermophilus* IDCC 2201. *S*. *thermophilus* IDCC 2201 was quantified in the fecal samples of G1, G2, and G3 with a standard curve (S2 Fig). The linearity range of the curve was 10^1^–10^8^ CFU/mL. The slope of the standard curve was -3.655 and the R^2^ value was 0.999, thus indicating high efficiency [36]. It was identified at a significantly higher level in G3 compared to G1 and G2 (p < 0.001) (Fig 7). The results of a previous study which administered *Lactobacillus brevis* DM9218, which has the effect of improving hyperuricemia, showed that *L*. *brevis* was detected in the feces of the DM9218-administered group, thus confirming a significant increase in the genus level [35]. In the current study, *S*. *thermophilus* IDCC 2201 was specifically detected at the strain level to verify whether *S*. *thermophilus* IDCC 2201 delivered to the rat intestine was actually effective.

## Conclusion

This study evaluated strains possessing genes related to purine metabolism as well as the ability to degrade purines; animal experiments involving the induction of hyperuricemia identified a probiotics strain that was able to both reduce blood uric acid levels and improve the intestinal environment. The selected *S*. *thermophilus* IDCC 2201 possesses an enzyme-related gene that decomposes hypoxanthine, and it was found to exhibit inosine and hypoxanthine degrading ability *in vitro*. Treatment with the selected strain was also shown to improve the serum uric acid concentration of hyperuricemia rats, and reproducibility was confirmed at a concentration of 1 × 10^9^ CFU/day. Another relevant consideration is that *S*. *thermophilus* IDCC 2201 changes microbial diversity and significantly increases SCFAs in the intestine. This was demonstrated to be ameliorated by an increase in *S*. *thermophilus* IDCC 2201 in fecal samples, thus demonstrating *S*. *thermophilus* IDCC 2201’s functions of reducing uric acid and strengthening intestinal health. Given the critical role played by gut microbiome composition in human health and disease through interactions between gut microbiota and human health, probiotics could represent a promising strategy when used as living therapeutics to prevent or treat disease. There is a need for metabolic studies to elucidate the mechanism by which *S*. *thermophilus* IDCC 2201 prevents hyperuricemia, and additional verification through clinical trials is also needed to continue developing it as a practical treatment.

## Supporting information

S1 Fig Hypoxanthine degradation analysis by HPLC. (TIF)

S2 Fig Real-time PCR standard curve for *S*. *thermophilus* IDCC 2201. (TIF)
