# Supplementary figures and images for: Evaluation of purine-nucleoside degrading ability and in vivo uric acid lowering of Streptococcus thermophilus IDCC 2201, a novel antiuricemia strain
Source: PLoS One. 2024 Feb 22;19(2):e0293378. doi: 10.1371/journal.pone.0293378 (PMC10883578; doi:10.1371/journal.pone.0293378)

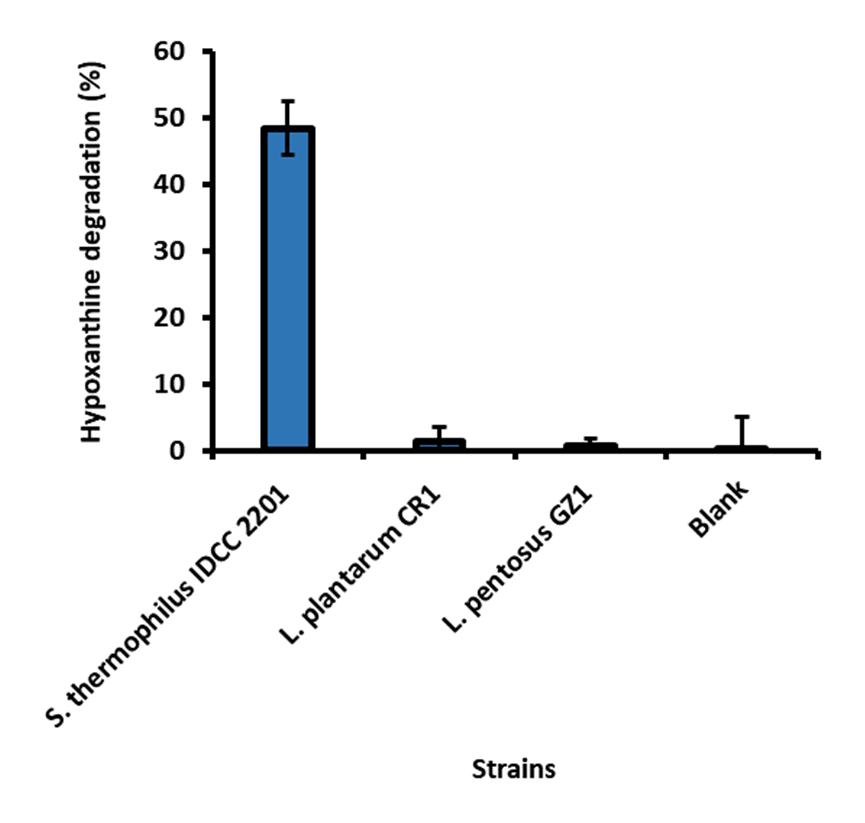

Supplement: S1 Fig — (TIF) [file pone.0293378.s001.tif]

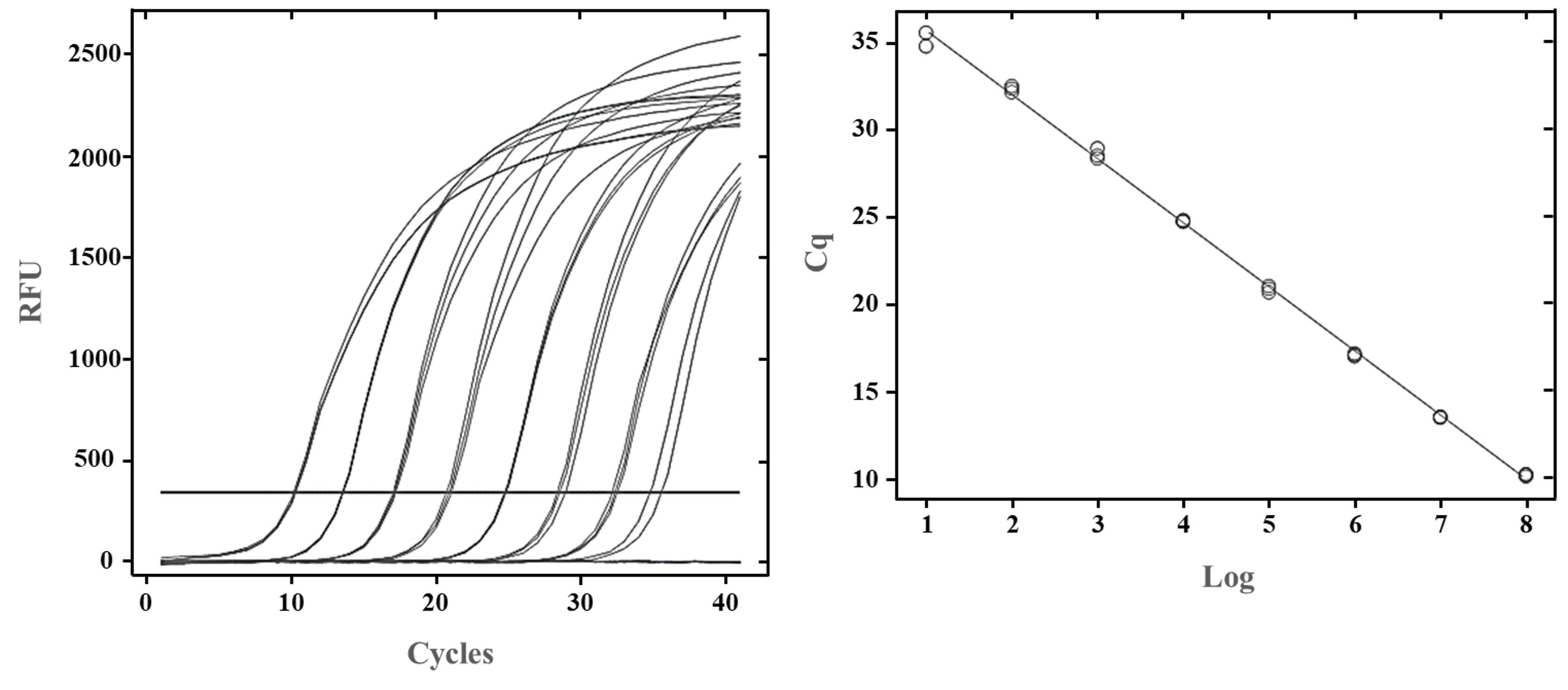

Supplement: S2 Fig — (TIF) [file pone.0293378.s002.tif]
